# Cultivar × Management Interaction to Reduce Lodging and Improve Grain Yield of Irrigated Spring Wheat: Optimising Plant Growth Regulator Use, N Application Timing, Row Spacing and Sowing Date

**DOI:** 10.3389/fpls.2020.00401

**Published:** 2020-04-28

**Authors:** Allan S. Peake, Kerry L. Bell, R.A. Fischer, Matt Gardner, Bianca T. Das, Nick Poole, Michael Mumford

**Affiliations:** ^1^Commonwealth Scientific and Industrial Research Organisation (CSIRO), Agriculture and Food, Toowoomba, QLD, Australia; ^2^Queensland Department of Agriculture and Forestry, Toowoomba, QLD, Australia; ^3^CSIRO Agriculture and Food, Canberra, ACT, Australia; ^4^Agricultural Marketing and Production Systems, Tamworth, NSW, Australia; ^5^The School of Agriculture and Food Sciences, The University of Queensland, St. Lucia, QLD, Australia; ^6^Foundation for Arable Research, Inverleigh, VIC, Australia

**Keywords:** G × E × M, wheat, irrigation, PGR, canopy management, in-crop N, crop duration

## Abstract

Severe lodging of irrigated spring-wheat in sub-tropical Australia has previously caused yield loss of between 1.7 and 4.6 t ha^–1^ (20–60% of potential yield). In response, agronomic management options were assessed for their ability to reduce lodging and increase grain yield, namely plant growth regulators (PGRs), timing of nitrogen (N) application, row spacing and sowing date, in combination with long and short duration cultivars across 15 irrigated environments from 2012 to 2016. Our study identified significant interaction between genotype, environment and agronomic management (G × E × M) for grain yield and lodging, although some combinations of agronomic techniques were broadly applicable across cultivars. PGR application improved grain yield of most cultivars in well-irrigated fields that had more than 120 kg ha^–1^ N (mineral N + fertiliser N) at sowing, with yield gains of up to 0.5 t ha^–1^ observed in both lodged and non-lodged fields. However, PGRs had little effect on grain yield when soil + fertiliser N at sowing was less than 80 kg ha^–1^ N. In-crop N application (compared to sowing N application) often improved grain yield of short duration, lodging resistant cultivars, but reduced the yield of long-duration, lodging susceptible cultivars in some environments. Narrow row spacing of 19 cm had the highest grain yield across cultivars in low lodging environments. At a severely lodged environment, narrow rows were the highest yielding for five out of six cultivars when PGRs were used, but was the highest yielding for only half of the tested cultivars when PGRs were not used. Cultivar × sowing date interaction for grain yield was also associated with the occurrence of lodging. Neither early nor late sowing had a consistent yield benefit across a range of cultivars, as lodging severity varied between sowing date depending on the timing of storm-induced lodging events. Lodging resistant long-duration cultivars had more stable grain yield across environments and increased grain yield in response to early sowing. Further research is needed to determine the optimum management strategy for new cultivars, because farmers do not always choose the most lodging resistant cultivars for reasons of cultivar disease resistance, grain quality and seed availability.

## Introduction

Irrigation of wheat on broad-acre farms in sub-tropical (i.e. between 23.5 and 31°S) eastern Australia has historically been uncommon due to the greater profitability of irrigated cotton (Hulugalle et al., 1999). Nevertheless, significant areas of irrigated spring-wheat were sown in the region in 2008, due to high grain prices and water availability. Unfortunately, substantial lodging occurred soon after anthesis in most production fields. Lodging related losses were estimated at 1.7 t ha^–1^ on average, with yield losses as high as 4.6 t ha^–1^ in extreme cases (Peake et al., 2014). Peake et al. (2016) found that high levels of soil nitrogen (N) and high seeding rates were probably responsible for the severe lodging experienced in 2008. These factors have previously been identified as increasing lodging risk in high-yielding winter and spring wheat production regions around the world (Stapper and Fischer, 1990; Easson et al., 1993; Sylvester-Bradley et al., 1997; Hobbs et al., 1998; Sylvester-Bradley et al., 2000; Berry et al., 2004).

Following these initial studies, additional research was conducted that aimed to decrease lodging and improve grain yield of irrigated wheat production systems in north-eastern Australia. Peake et al. (2014, 2016) identified traditional long duration cultivars as being more susceptible to lodging and lower yielding than short duration cultivars in the region. Subsequently, Peake et al. (2018) demonstrated that newly released long duration cultivars had a consistent yield benefit in comparison to short duration cultivars, when cultivars were sown at different times to achieve synchronised anthesis during the optimal anthesis window. However, the long duration cultivars were still more prone to lodging than short duration cultivars, and improved agronomic management is therefore still needed to minimise lodging in the region.

Several agronomic management options often referred to as canopy-management practices (Sylvester-Bradley et al., 1997; Sylvester-Bradley et al., 2000) are known to reduce lodging risk and severity. Reduced light quality and quantity (i.e. increased shading) has been shown to weaken the stem base and surface roots, thus increasing lodging risk (Sparkes and King, 2008; Sparkes et al., 2008). Avoiding excessive canopy development during vegetative growth has been shown to reduce lodging risk without reducing grain yield (Sylvester-Bradley et al., 2000; Peake et al., 2016). Reducing crop height also reduces lodging risk by reducing leverage applied to the stem base during windstorms (Baker et al., 1998; Berry et al., 2003). Canopy management practices used to reduce lodging around the world include plant growth regulator (PGR) application (Herbert, 1982; Knapp et al., 1987; Crook and Ennos, 1995; Tripathi et al., 2003), in-crop N application (Mulder, 1954; Kheiralla et al., 1993; Crook and Ennos, 1995; Berry et al., 2000; Islam et al., 2002; Tripathi et al., 2003; Peake et al., 2016; Wu et al., 2019), wider row spacing (Stapper and Fischer, 1990; Din et al., 2017) and delayed sowing (Hanley et al., 1961; Stapper and Fischer, 1990; Berry et al., 2000; Spink et al., 2000).

Few studies have been conducted to assess the suitability of these practices for broad-acre irrigation farms in sub-tropical Australia. Peake et al. (2016) determined that the optimal N management strategy for a representative Vertosol soil (Isbell, 2016) was for the soil to contain 50–70 kg N/ha (mineral N + sowing fertiliser N) at sowing, with the remainder of the crop N requirement applied during the cropping season. This strategy induced visible N stress during tillering which reduced vegetative growth prior to in-crop N application at floral initiation and flag leaf emergence, and subsequently achieved a significant reduction in lodging. However, their study used two outdated cultivars that were subsequently assessed as having moderate to high levels of lodging susceptibility, and was only conducted across two seasons in a single environment. No research has been conducted on the ability of row spacing or PGRs to control lodging in conjunction with the range of cultivars available to farmers in the region. And while the previously mentioned study of Peake et al. (2018) demonstrated the benefits of long duration cultivars for irrigated wheat production in the region, early sowing is known to cause increased lodging risk in high yielding wheat production regions such as Europe and New Zealand. In these environments, late sowing is promoted as a lodging control method for high risk (e.g. high soil fertility) conditions.

This study extends the study of Peake et al. (2018) and reports the findings of a long-term cultivar × agronomy research program, which aimed to identify the optimum agronomic management practices for cultivars adapted to irrigated, broad-acre spring-wheat production regions of sub-tropical Australia.

## Materials and Methods

Experiments were conducted at multiple locations from 2012 to 2016. Table 1 details experiments conducted to investigate the interaction of cultivar with PGR application, while Table 2 details experiments examining the interaction of N application timing, row spacing and sowing date. Due to the large number of cultivars and agronomic treatments, factorial experiments did not include all combinations of cultivar and agronomic management at each location. The majority of experiments were conducted at Spring Ridge (31.3871°S; 150.2469°E) and Gatton (27.54°S; 152.33°E), chosen for their representation of two environmental extremes within the target population of environments. Spring Ridge is a cooler, higher latitude environment with a longer growing season and high yield potential, while Gatton is a warmer, lower latitude environment with a shorter growing season and moderate yield potential (Peake et al., 2014, 2016). Additional locations were Narrabri (30.3324° S; 149.7812° E), which has a slightly shorter growing season than Spring Ridge, and Emerald (23.5273° S; 148.1646° E) near the Tropic of Capricorn and having a shorter growing season than Gatton.

**TABLE 1:** Summary of experiment details, factor entries and PGR effect on grain yield and lodging for PGR × cultivar experiments between 2012 and 2016.

Experiment details^1^				Factor entries					PGR effect on grain yield			Grain yield (t ha^–1^)		Grainfill lodging (%)	
					
Lodging severity	Location and season	Cultivar duration	Sowing N status	PGR	Yr	DG	Cv	PGR factor significance (Grain Yield)	Positive	NS	Negative	Expt. Mean	PGR effect (min, mean, max)	Expt. Mean	PGR effect (min, mean, max)
**High sowing N experiments**															
No lodging	Em12	Short	High	2	1	1	16	N.S.	0	16	0	5.64	NA	Nil	Nil
No lodging	SR15/16	Long	High	2	2	1	4	Cv × PGR	3	1	0	8.22	(0, 0.68, 1.16)	Nil	Nil
No lodging	SR15/16	Short	High	2	2	1	8	Cv × PGR	6	2	0	7.89	(0, 0.40, 0.71)	Nil	Nil
Negligible	Brz13	Long/short^2^	High	2	1	2	36	Main effect	36	0	0	5.42	0.56	1.9	(−5.3, −1.0, 0)
Negligible	Em13	Short	High	2	1	1	12	Cv × PGR	2	9	1	6.56	(−0.6, 0.2, 0.91)	1.2	(−5.3, −1.3, 0)
Negligible	Nar13	Long	High	2	1	1	16	Cv × PGR	1	15	0	6.60	(0, 0.13, 1.48)	2.5	(−13.9, −1.3, 6.4)
Negligible	Gat15/16	Short	High	2	2	1	6	Cv × PGR	2	4	0	6.75	(0, 0.06, 0.39)	2.1	(−0.8, −0.1, 0)
Moderate	Brz12	Long	High	2	1	1	22	Main effect	22	0	0	6.81	0.41	7.1	NA
Moderate	Nar13	Short	High	2	1	1	16	N.S.	0	16	0	6.85	NA	6.9	(−3.2, −1.3, 0)
Severe	Gat13	Short	High	2	1	1	15	Main effect	15	0	0	5.79	0.52	11.9	(−28.3, −12.3, 0)
**Moderate sowing N experiments**															
Negligible	Em13	Short	Moderate	2	1	1	12	Cv × PGR	2	9	1	6.56	(−0.6, 0.2, 0.91)	1.2	(−5.3, −1.3, 0)
Negligible	SR15/16	Long	Moderate	2	2	1	4	Cv × PGR	2	2	0	8.22	(0, 0.2, 0.54)	Nil	Nil
Negligible	SR15/16	Short	Moderate	2	2	1	8	Cv × PGR	4	3	1	7.89	(−0.5, 0.28, 0.6)	Nil	Nil
Negligible	Gat16	Short	Moderate	2	1	1	3	Cv × PGR	0	3	0	6.75	(0, 0, 0)	2.1	NA
Severe	SR14	Long/short^2^	Moderate	2	1	2	12	Cv × PGR	8	3	1	7.17	(−0.53, 0.41, 0.87)	24.5	(−10.6, −3.0, 4.6)
**Low sowing N experiments**															
Negligible	Brz13	Long	Low	2	1	1	18	N.S.	0	18	0	5.35	NA	1.2	(−1.6, −1.3, −1.0)
Negligible	Brz13	Short	Low	2	1	1	18	Cv × PGR	3	15	0	5.48	(0, 0.3, 1.1)	0.4	(−0.7, −0.5, −0.3)
Negligible	Gat15	Short	Low	2	1	1	3	Cv × PGR	0	3	0	6.75	(0, 0, 0)	2.1	NA
Mild	Brz12	Long	Low	2	1	1	22	N.S.	0	22	0	6.92	NA	4.8	NA
**Partially irrigated experiments**															
Negligible	Brk13	Long	Moderate	2	1	1	12	Main effect	0	0	12	4.80	−0.21	Nil	Nil
Negligible	Brk13	Short	Moderate	2	1	1	12	N.S.	0	12	0	5.15	NA	Nil	Nil

*PGR = plant growth regulators, Yr = year (or season), DG = duration group (i.e. grouped long and short duration cultivars sown on different sowing dates), Cv = cultivar, Em = Emerald, SR = Spring Ridge, Nar = Narrabri, Brza = Breeza, Gat = Gatton, Brk = Brookstead, N.S = no significant effect (*p* > 0.05), Cv × PGR: significant interaction was observed between cultivar and P GR treatment (*p* < 0.05), main effect: the main effect of PGR was significant (*p* < 0.0 5) and no higher order interactions involving PGR were significant (*p* > 0.0 5). ^1^ = Each line of the table represents a single statistical analysis conducted at a single location, with multi-year and duration group factors also listed where relevant. ^2^ = Cultivar was nested within duration group for these experiments.*

**TABLE 2:** Summary of experiment details, factor entries and results summary for grain yield and lodging, for the N timing, sowing date and multi-factor row-spacing experiments between 2014 and 2016.

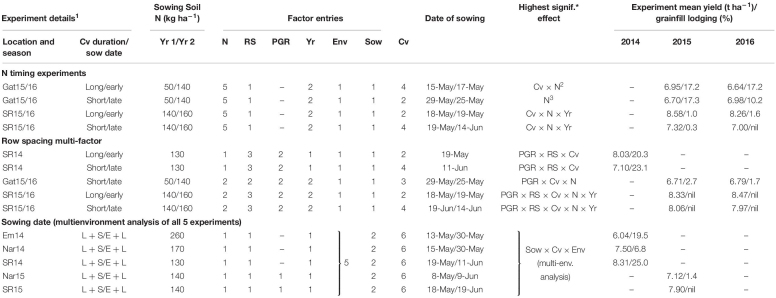

Cv = cultivar, Yr = year, N = nitrogen, RS = row spacing, PGR = plant growth regulator, Env = environments (i.e. location × year combinations), Sow = sowing dates, L + S = long and short, E + L = early and late, SR = Spring Ridge, Em = Emerald, Nar = Narrabri, Gat = Gatton. * = Significant at *p* < 0.05 unless otherwise stated. ^1^Each line of the table represents a single statistical analysis conducted at a single location, except where indicated otherwise. ^2^*p* = 0.056, ^3^*p* = 0.086.

### Agronomic Treatments

A wide range of germplasm was screened in initial experiments (2012 and 2013) before conducting multi-factor experiments with a smaller set of high-performing cultivars from 2014 to 2016. When comparing cultivars in combination with three of the agronomic treatments investigated (PGRs, N application timing and row spacing), cultivars were sown on one of two available sowing dates (i.e. early or late) as recommended for each cultivar in that specific location, to allow them to reach anthesis approximately during the optimum flowering window for each location. The cultivars LRPB Cobra and LRPB Trojan (hereafter referred to as Cobra and Trojan) were classified as short duration cultivars at the cooler southern environment (Spring Ridge) but as long duration cultivars at the warmer environment (Gatton). All the cultivars discussed within this study are protected by Plant Breeders Rights legislation within Australia. Other agronomic treatments consisted of combinations of the following.

#### Plant Growth Regulators (PGRs)

The PGR treatment consisted of 1000 ml/ha chlormequat chloride mixed with 200 ml/ha trinexapac-ethyl, applied approximately at GS31 (Tottman, 1987). This PGR-mix was applied on the same day for all cultivars in each experiment, hence the application occurred approximately when mean crop stage of all cultivars was GS31. Variation in crop development between cultivars thus meant that the application was not applied precisely at GS31 for each cultivar in all experiments. A control treatment (i.e. no PGR applied) was also included for all cultivars, in all PGR experiments.

#### N Application Timing

Two N strategies (sowing N and in-crop N) were compared for their effect on grain yield and lodging. The aim of the in-crop N application strategy was to apply no fertiliser N [other than small quantities of starter fertiliser such as mono ammonium phosphate (MAP)] until GS31 (Tottman, 1987). This involved surface-spreading of urea to ensure that the crop had been supplied with 200 kg/ha N (taking both soil mineral N at sowing and fertiliser N into account) by GS31. The remainder of the N fertiliser required to achieve target yield potential was applied at GS39. The in-crop N strategy was compared with an alternate ‘sowing N’ strategy, where all N was applied either prior to, or within 2 weeks of sowing. Three other in-crop N strategies were also tested which created five N treatments in total; however, only the two described above are reported herein. Total season N supply ranged from 275 to 400 kg N ha^–1^ depending on location and potential yield. This strategy achieved non-limiting N status through grainfilling as evidenced by the grain protein being in excess of 13% in all experiments (Goos et al., 1982; Holford et al., 1992).

#### Row Spacing

A range of row spacing was tested at multiple locations, with the exact spacing depending on the capability of local sowing equipment. Typically, the wide row spacing was 38 cm, the intermediate spacing was 25 or 28 cm and the narrow row spacing was 19 cm.

#### Sowing Date and Cultivar Duration

Six cultivars were compared on both an early and late sowing date in 2014 and 2015, at three locations (Emerald, Narrabri and Spring Ridge) to determine whether late sowing could be used to reduce lodging risk and increase grain yield. The cultivars were Cobra, Trojan, Kennedy, EGA Bellaroi (hereafter referred to as Bellaroi), Caparoi and Suntop. Sowing dates were approximately 2–3 weeks apart (Table 2).

Due to the importance of sowing on time and the requirement for a sowing irrigation at some locations, sowing date treatments were sown in adjacent but separate areas. This avoided the problem experienced by Peake et al. (2016) in which late sown areas of split plot experiments were irrigated on the first sowing date, subsequently experienced rainfall, and were then too wet to sow on the optimal late sowing date thus preventing synchronisation of anthesis.

### Plot Management and Statistical Analysis

Plots at Narrabri, Breeza, Emerald and Gatton were 2 m wide × 7 m long and trimmed to 5 m long at harvest. Longer, narrower plots 1.4 m wide × 12 m long were sown at Spring Ridge and trimmed to 10 m at harvest. Seeding rate was 110 seeds per m^2^. Edge rows were not trimmed due to the similarity of lateral plot dimensions with the 2 m bed configuration commonly used on furrow-irrigated farms within the region. The gap between outside rows of neighbouring plots was 50 cm at all locations except Narrabri where it was 60 cm. Yield was calculated by multiplying final plot length by the distance between the centre of neighbouring plots, and grain yield is reported at 12% moisture. Pests and diseases were effectively controlled using a range of agrochemicals as preferred by local co-operators, except for a powdery mildew (*Blumeria graminis*) outbreak that became noticeable during late grain filling at Narrabri in 2014.

Overhead irrigation systems (i.e. lateral move irrigators or hand-shift sprinklers) were used for all experiments except those conducted at Breeza, where experiments were furrow irrigated. Irrigation scheduling was timed to avoid water stress by applying irrigation weekly to fully replace crop evapotranspiration (locally known as ‘full irrigation’). The effectiveness of implementation varied between locations and seasons due to climate variation (i.e. evaporative demand and rainfall), logistical issues and individual soil characteristics. The experiments at Brookstead 2013 were ultimately water-stressed due to an unexpectedly reduced supply of irrigation water and are thus referred to as ‘partially irrigated’ experiments.

Lodging was calculated as ‘average grainfill lodging’ as described by Peake et al. (2016). This involved rating lodging where possible on the first day after each potential lodging event (rainfall or irrigation), and every 5–7 days between lodging events. Lodging score for a given day were similar to those used by Mulder (1954), i.e. the average stem angle from vertical (divided by 90 and expressed as a %) for the whole plot and ranged from 0% (no lodging) to 100% (completely lodged). These data were then used to calculate the average lodging during grainfill (also referred to as ‘grainfill lodging’) by multiplying each daily score by the number of days before the next score was taken, and then averaging these over the number of days between anthesis and harvest. This method quantifies the likelihood that lodging may have caused physiological disruption to the crop. By contrast, the lodging score at harvest (Stapper and Fischer, 1990) may be wholly due to a single, late lodging event immediately before harvest, and not reflect on the development of lodging through the season.

Experiments were generally arranged as randomised complete block designs incorporating a latin square design to avoid the same treatments occurring in the same row/column. The exception was at the Narrabri 2014 sowing date experiments (Table 2) which were implemented as split-plot experiments, with the agronomic treatments randomly allocated at the main plot level and cultivars at the sub-plot level. All experiments had three replications for each combination of cultivar, sowing date and agronomic treatment.

Combined experiment analyses were conducted using the REML (residual maximum likelihood; Patterson and Thompson, 1971) procedure in GENSTAT (19th Edition, VSN International, 2017), using linear mixed models with individual trial designs and separate residual variances fitted for each trial. Location and season were considered random effects, while agronomic treatments and cultivars were fitted as fixed effects. Square-root transformation was necessary before analysis of average lodging during grainfill for some experiments, and the results reported have been back-transformed. In all analyses, the level of significance was set at *P* = 0.05 unless stated otherwise. The least significant difference (LSD) procedure was used to compare levels of an effect if the *F*-test was significant.

## Results

### Interaction of Cultivars With Plant Growth Regulator

Significant grain yield increases were observed in most of the PGR × cultivar experiments (Table 1). Significant interactions between cultivar and PGR for grain yield meant that no specific cultivar consistently achieved increased grain yield in response to PGR application. Out of 251 comparisons from the fully irrigated experiments conducted using a range of cultivars, locations, seasons and sowing N availability, 106 showed a yield advantage associated with PGR application, 141 were not significantly different, and 4 showed a significant yield decrease. Cultivars that showed a significant yield decrease at Emerald 2013 (Merinda), Spring Ridge 2014 (Lancer) and Spring Ridge 2015/2016 (Cobra) all achieved a significant positive yield response to PGRs in at least one other experiment. All cultivars used across multiple experiments had a significant increase in grain yield in response to PGR application in at least one experiment. In the two partially irrigated experiments at Brookstead in 2013, PGRs did not increase grain yield of the short-duration cultivars, and significantly decreased grain yield by 0.21 t ha^–1^ in the long duration cultivars (Table 1).

**FIGURE 1:**
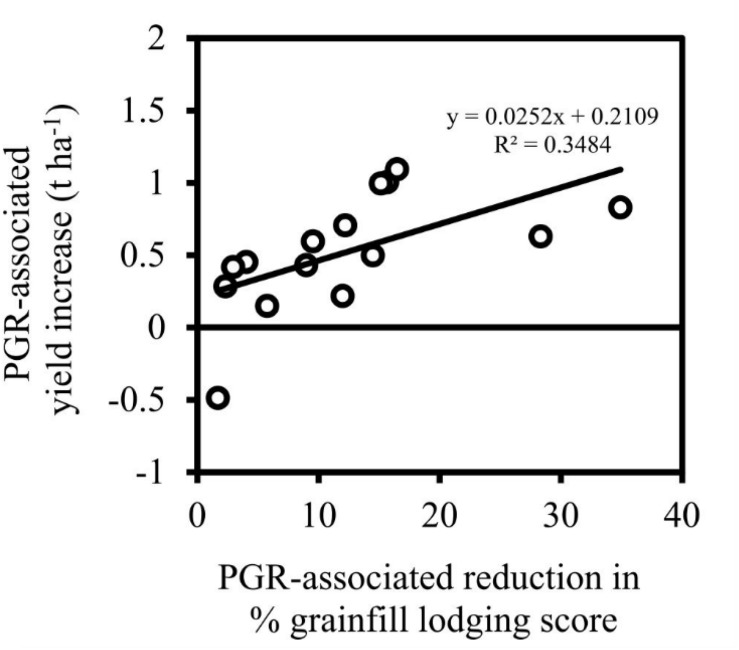
Grain yield increase associated with PGR-mix application (i.e. the difference between predicted grain yield of PGR treated and control plots of the same cultivar) vs. reduction in grainfill lodging score (i.e. the difference between grainfill lodging score of PGR treated and control plots for the same cultivar) of the equivalent plot comparisons, for 15 cultivars tested at Gatton in 2013. The solid line represents the significant (*F*_prob_ = 0.021) linear regression.

**FIGURE 2:**
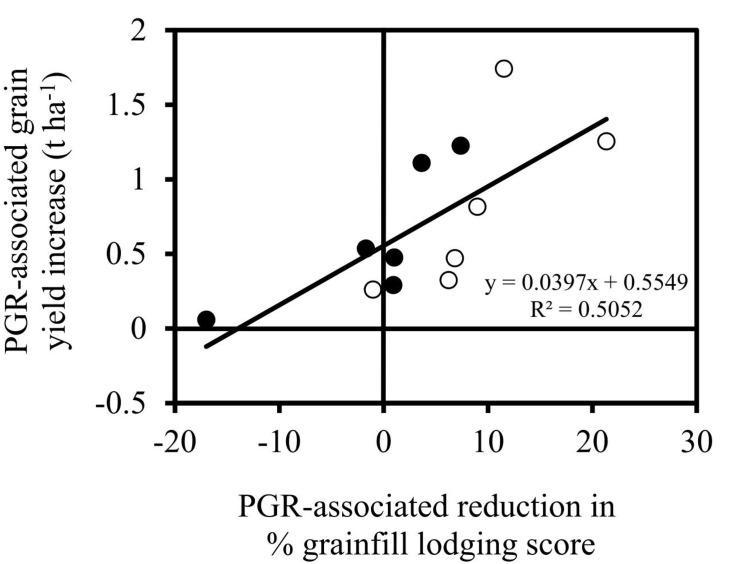
Grain yield increase associated with PGR-mix application (i.e. the difference between predicted grain yield of PGR treated and control plots of the same cultivar) vs. reduction in grainfill lodging score (i.e. difference between grainfill lodging score of PGR treated and control plots for the same cultivar) of the equivalent comparisons, across six cultivars and two sowing dates at Spring Ridge in 2014. The solid line represents the significant (*F*_prob_ = 0.01) combined linear regression of data from early (∙) and late (∘) sown experiments.

The benefits of PGR application were most clearly demonstrated at Gatton in 2013, where a significant positive relationship was observed between the PGR-associated yield gain and lodging reduction, across a range of cultivars (Figure 1). The maximum PGR-associated yield gain for an individual cultivar in this experiment was 1.1 t ha^–1^. However, PGR application did not always reduce grainfill lodging and even increased lodging occasionally, as seen at Spring Ridge 2014 (Figure 2) where a significant linear trend was also observed between lodging reduction, and the yield increase attributed to PGR application. In this experiment the *x*-intercept of the regression line was −15, with one cultivar lodging more severely in response to PGR application, and several cultivars having yield increases of 0.25–0.5 t ha^–1^ in association with PGR application despite having no decrease in lodging. The maximum PGR-associated yield gain for an individual cultivar in this experiment was 1.75 t ha^–1^.

**FIGURE 3:**
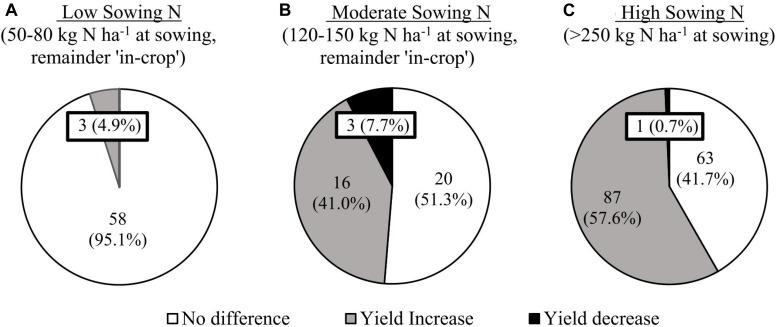
Pie chart showing PGR response (significant grain yield increase, no change, significant grain yield decrease) across cultivar comparisons grouped into **(A)** low, **(B)** moderate and **(C)** high sowing N fields. Results collated from all experiments listed in Table 2 except for the partially irrigated experiments at Brookstead 2013.

The probability of observing PGR-associated grain yield increases was greatest in the fully irrigated experiments where sowing N status was high or moderate (Table 1 and Figure 3). Nearly 60% of cultivar × PGR comparisons displayed a significant grain yield increase when sowing N (i.e. mineral N + fertiliser N) was greater than 250 kg ha^–1^, and 41% had a significant grain yield increase when sowing N was between 120 and 150 kg ha^–1^. No significant difference was observed between the PGR and control treatments for most remaining cultivar × PGR combinations in experiments with high or moderate sowing N status. A negative grain yield response to PGR application was observed in three out of 39 comparisons in the moderate sowing N experiments, and one of the 151 comparisons in the high sowing N experiments. At low sowing N experiments (where between 50 and 80 kg N ha^–1^ was available from sowing for vegetative growth), only 5% of cultivar × PGR comparisons achieved a significant yield increase in response to PGR application, while the remainder (95%) had no significant difference in grain yield between PGR treatments (Figure 3). A chi-squared test confirmed that the ratio of positive, non-significant and negative grain yield differences between PGR × cultivar combinations was significantly different between the high, moderate and low sowing N fields (data not shown).

Gatton 2013 and Spring Ridge 2014 possessed high and moderate sowing N, respectively, and were the two most heavily lodged experiments. These experiments contained two of the biggest grain yield increases associated with PGR application across a range of cultivars (0.52 and 0.41 t ha^–1^ respectively; Table 1). Yet PGR application also increased grain yield by approximately 0.5 t ha^–1^ in some experiments with high or moderate sowing N when lodging was negligible, e.g. Breeza 2013, Spring Ridge 2015 and 2016 (Table 1).

### Interaction of Cultivars With N Application Strategy

**FIGURE 4:**
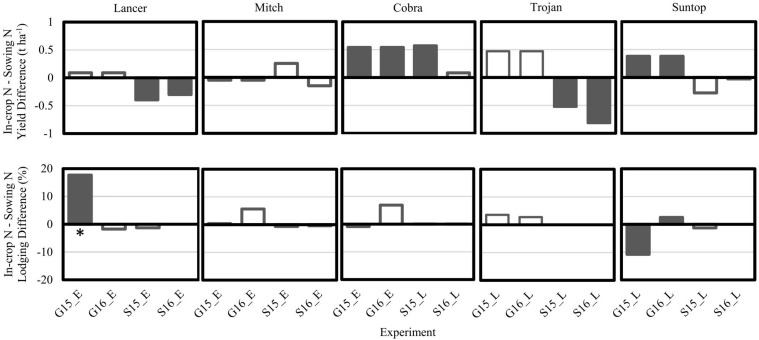
Difference between in-crop N application and sowing N application strategies for grain yield and lodging score for the five cultivars sown at both the Gatton (G) and Spring Ridge (S) sites in the early (E) or late (L) sown N timing × Cultivar experiments in 2015 and 2016. Filled bars indicate that the difference between N treatments was significantly different (*p* < 0.05); empty bars were not significantly different (*p* > 0.05). A positive value indicates larger absolute values for the in-crop N treatment, meaning that bars above the line in the lodging graphs demonstrate more severe lodging associated with the in-crop N treatment. *Lodging for Lancer at Gatton 2015 began first in the sowing N treatment, but stems straightened to the extent that average grainfill lodging was ultimately worse in the in-crop N treatment which lodged heavily later in the season.

Significant N treatment effects were observed either as main effect or as interactions with cultivar and/or season for grain yield and lodging in the cultivar × N timing experiments conducted at Spring Ridge and Gatton in 2015 and 2016 (Table 2 and Figure 4). Only results for cultivars that were included both at Spring Ridge and Gatton are reported. Suntop and Cobra both achieved significantly increased yield of 0.4–0.6 t ha^–1^ in conjunction with in-crop N application at Gatton in both seasons, but only Cobra yielded significantly more at Spring Ridge in 2015. Mitch did not have a significantly different yield between N treatments in any of the experiments. Grain yield of Lancer and Trojan was significantly decreased in response to in-crop N application in both seasons at Spring Ridge (by 0.3–0.4 t ha^–1^ for Lancer and 0.5 to 0.8 t ha^–1^ for Trojan), but was not significantly different between N treatments at Gatton.

The significant yield increases associated with in-crop N application for Cobra and Suntop were not accompanied by significant reductions in lodging, with the exception of Suntop at Gatton in 2015 (Figure 4). Surprisingly, lodging in Lancer was significantly worse in association with in-crop N application at Gatton in 2015. Close examination of the timing of lodging revealed that the Lancer sowing N treatment lodged before the in-crop N treatment, but the stems then straightened in a phototropic response. The in-crop N plots lodged later but did not recover as well due to the later growth stage, leading to the greater average lodging score during grainfilling.

**FIGURE 5:**
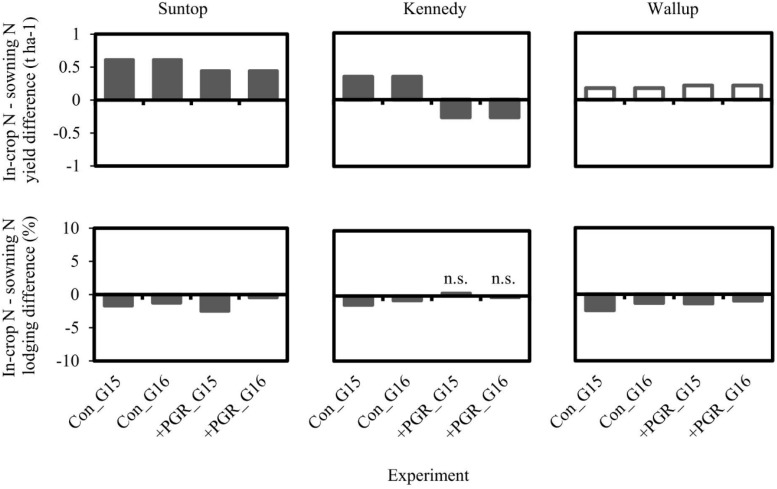
Difference between in-crop N application and sowing N application strategies for grain yield and lodging score for three short duration cultivars in the N timing × row spacing multi-factor experiments from Gatton (G) in 2015 and 2016. A positive value indicates larger absolute values for the in-crop N treatment, meaning that bars above the line in the lodging graphs demonstrate more severe lodging associated with the in-crop N treatment. Filled bars were significantly different (*p* < 0.05) and empty bars were not significantly different (*p* > 0.05). ‘Con’ = Control (i.e. no PGRs applied), ‘ + PGR’ = PGR mix applied. ‘n.s.’ = not significantly different (*p* < 0.05).

In a second experiment conducted using short duration cultivars at Gatton, significant interaction was observed between cultivar, PGR treatment and N application strategy for grain yield and lodging. The short duration cultivars in this experiment exhibited different responses to PGR and N treatments (Figure 5). Grain yield of Suntop was significantly increased in response to in-crop N application compared to sowing N application (by approximately 0.5 t ha^–1^) regardless of whether PGRs were applied, while grain yield of Wallup was not significantly different between N treatments in combination with either PGR treatment. In-crop N application increased grain yield of Kennedy compared to sowing N application when no PGRs were applied (0.35 t ha^–1^), but decreased grain yield of Kennedy (−0.27 t ha^–1^) when combined with PGR application. Lodging was significantly reduced for in-crop N compared to sowing N for all three cultivars when no PGRs were applied, and also for Suntop and Wallup when PGRs were applied.

### Interaction of Cultivars With Row Spacing, PGRs and N Timing

**FIGURE 6:**
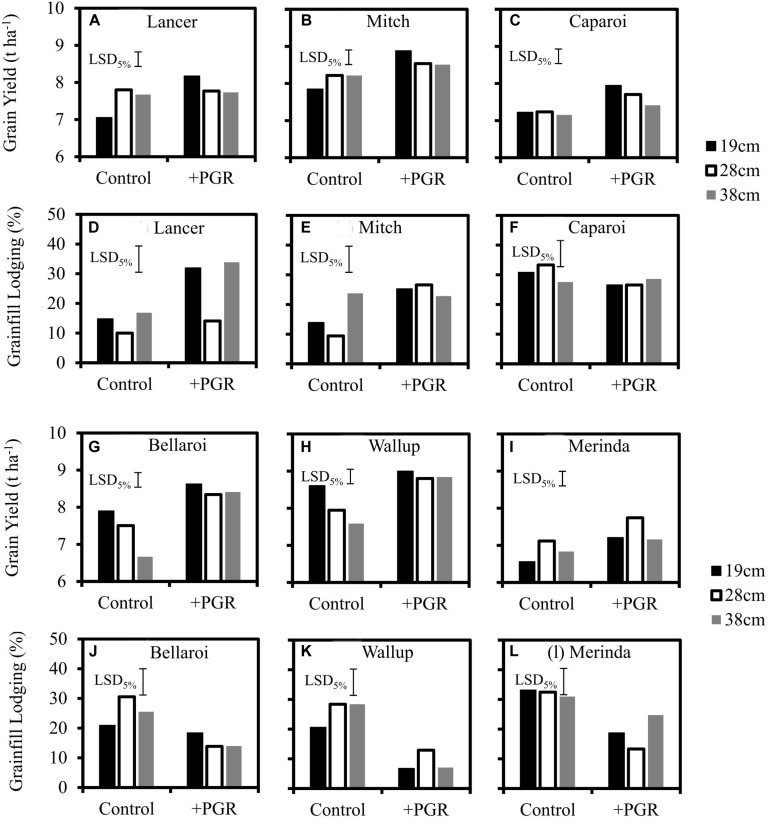
Grain yield and grainfill lodging for three row spacings in response to plus and minus PGR-mix treatments in six cultivars sown on their optimum sowing date at Spring Ridge in 2014. LSD_5__%_ represents the average LSD across all pairwise comparisons. Maximum LSD was 5% greater than average LSD, while minimum LSD was 10% below average LSD. **(A–F)** Grain yield and lodging for Lancer, Mitch, Caparoi. **(G–L)** Grain yield and lodging for Bellaroi, Wallup, Merinda.

Significant cultivar × PGR × row spacing interaction for grain yield was observed at Spring Ridge in 2014 (Table 2 and Figure 6) where 130 kg ha^–1^ of N was available at sowing and severe lodging occurred. The long duration cultivars Lancer and Mitch both displayed similar interactions of row spacing and PGRs, with the narrowest row spacing (19 cm) being the highest yielding of all row spacing treatments when PGRs were applied, but the lowest yielding when PGRs were not applied (Figures 6A,B). These trends were not directly associated with severity of lodging (Figures 6D,E) as lodging was more severe in conjunction with PGR application for these two cultivars. The short duration cultivar Caparoi also had increasing yield with narrower row spacing when PGRs were applied (Figure 6C) but the intermediate row spacing exhibited the greatest yield in the absence of PGR application. Lodging in Caparoi was not significantly different between PGR and control plots (Figure 6F).

Bellaroi and Wallup exhibited different yield response patterns across row spacings (Figures 6G,H) compared to Lancer and Mitch at Spring Ridge in 2014. Narrow row spacing gave a small (non-significant) yield increase when PGRs were applied, but a large, significant yield increase when PGRs were not applied that was associated with reduced lodging in the narrow row spacing (Figures 6J,K). Bellaroi and Wallup had substantially less lodging when PGRs were applied on average across row spacings (Figures 6J,K). Merinda (Figures 6I,L) exhibited different yield response patterns across row spacings to the other cultivars, with the intermediate row spacing (25 cm) being the highest yielding regardless of whether PGRs were used. Despite the significant interactions with row spacing and cultivar, PGR application was generally associated with significantly greater grain yield compared to the untreated control across the range of cultivars (Figure 6), in agreement with the experiments reported in Section ‘Interaction of Cultivars With Plant Growth Regulator’.

At Gatton in 2015 and 2016 where only mild lodging occurred, there was a near-significant higher order interaction (*p* = 0.057) of row spacing with season, PGR application and cultivar for grain yield. Grain yield was not significantly different between wide and narrow row spacing for 10 out of the 12 comparisons (data not shown). However, grain yield was significantly greater in the wider (35 cm) row spacing by 0.16 t ha^–1^ for the cultivar Suntop in 2015 when no PGRs were applied, and also by 0.34 t ha^–1^ for the cultivar Kennedy in combination with PGR application in 2016. Grain yield trends across treatments were not associated with treatment differences for lodging within this experiment.

**FIGURE 7:**
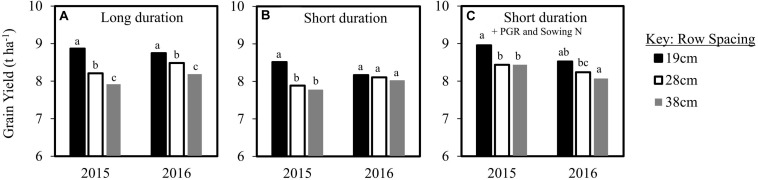
Grain yield of row spacing treatments from a combined analysis of Spring Ridge across the low-lodging seasons of 2015 and 2016 for **(A,B)** long and short duration cultivars on average across PGR and N treatments, and **(C)** short duration cultivars in combination with the site-specific highest yielding management practices of sowing N application in combination with application of the PGR-mix.

Significant grain yield increases were observed in conjunction with narrow row spacing at Spring Ridge in 2015 and 2016 (Figure 7), where negligible lodging was experienced in both seasons. A significant five-way interaction of cultivar, row spacing, N regime, PGR treatment and seasons was observed for grain yield within both the long and short duration cultivar groups (Table 2). The significant five-way interaction was predominantly exhibited as variability between the low yielding agronomic factor combinations (data not shown) that are less favoured by growers in the region (e.g. no PGRs applied). In particular, in 2016 when in-crop N application was used and PGRs were not applied, both Cobra and Suntop had a reverse trend where grain yield decreased with narrower row spacing. This trend was isolated as it was not evident in the previous season, or within the same season when PGRs were used.

On average across PGR and N treatments, significant grain yield increases were observed in conjunction with narrow row spacing at Spring Ridge in 2015 and 2016 (Figure 7). In particular, the 19 cm row spacing showed a 0.7–0.8 t ha^–1^ grain yield increase compared to the 38 cm row spacing, on average across the long duration cultivars (Lancer and Mitch) in both seasons and the short duration cultivars (Bellaroi, Cobra, Trojan and Suntop) in 2015 (Figures 7A,B). The highest grain yields were achieved at Spring Ridge by combining the 19cm row spacing with PGR application and sowing N application (Figure 7C).

### Interaction of Cultivar and Crop Duration With Sowing Date and Environment

**FIGURE 8:**
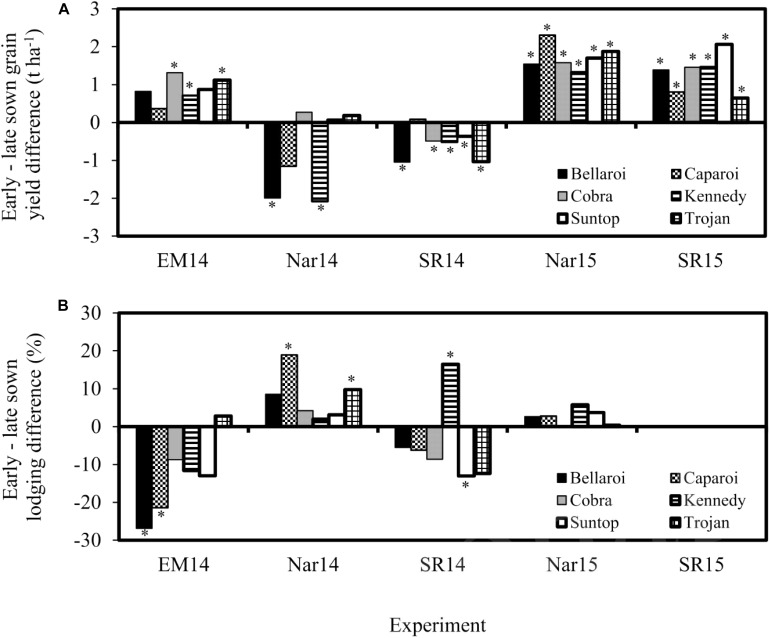
Difference between early and late sown grain yield **(A)** and lodging score **(B)** of six cultivars sown at 5 environments in 2014 and 2015 (EM = Emerald, Nar = Narrabri, SR = Spring Ridge). A positive value indicates larger absolute values for the early sowing treatment, meaning that bars above the line in the lodging graphs demonstrate more severe lodging associated with early sowing. An asterisk ‘*’ signifies that the difference between early and late sowing was significant (*p* < 0.05).

**FIGURE 9:**
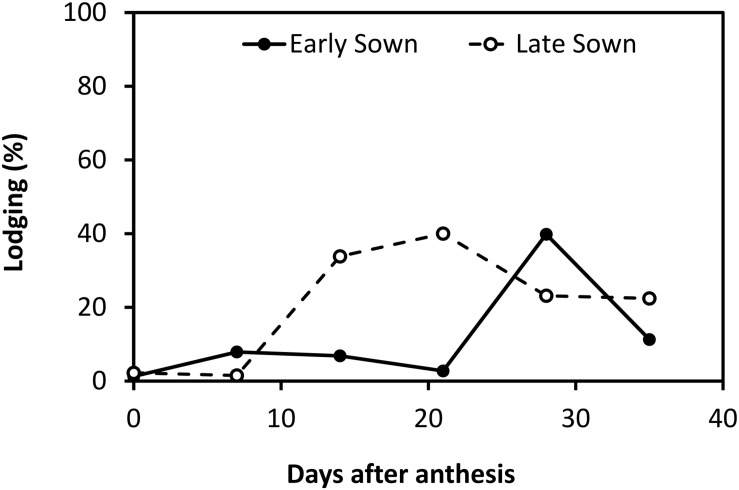
Mean lodging score vs. days after anthesis from the early (∙) and late (∘) sown experiments at Emerald in 2014. A 14-day difference was recorded between the date of 50% anthesis for the two sowing dates, hence storm-related lodging was experienced at an earlier growth stage in the late sown experiment.

Significant cultivar × sowing date × environment interaction was observed for grain yield (Table 2 and Figure 8) when comparing six cultivars across two sowing dates and five environments. The early sowing date achieved the highest grain yields at three environments (Emerald 2014, Narrabri 2015 and Spring Ridge 2015) while the late sowing date had the highest yields at two environments (Narrabri 2014 and Spring Ridge 2014). Lodging was more severe on the early sowing date at Narrabri 2014 and on the late sowing date at Emerald 2014, potentially contributing to the yield difference at these environments. At Spring Ridge 2014 the highest yielding sowing date (late sowing) also experienced the greatest lodging. Lodging at this location was initially worse on the early sowing date, but a severe late lodging event affected the late sown experiments more than the early sowing (data not shown). At Emerald 2014, the increased lodging associated with late sowing was probably related to the timing of storms. These caused more severe lodging in the late sown experiment in comparison to the early sown experiment (Figure 9).

Cultivar × sowing date interaction was more apparent at Narrabri 2014. Three cultivars (Cobra, Suntop and Trojan) had similar grain yield between the sowing dates, while the remaining cultivars had substantially decreased grain yield (>1.0 t ha^–1^) on the early sowing date. The largest and most consistent yield increases associated with early sowing were at Narrabri and Spring Ridge in 2015. As discussed by Peake et al. (2018), the early sown treatments at these locations probably experienced less water stress during grainfilling due to the development of deeper root systems. This stress occurred during heat wave conditions, when irrigation infrastructure could not supply enough water to equal potential evapotranspiration.

## Discussion

The results of the study showed that G × E × M (genotype by environment by management interaction) is present within irrigated, sub-tropical wheat production systems. Some agronomic practices (PGRs and narrow row spacing) generally improved grain yield across a wide range of applicable cultivars and environments, particularly when used together. Other practices (in-crop N application and early/late sowing) increased grain yield for specific cultivar, management or environment combinations. Interaction was also observed between multiple management practices, and it is important to understand the specific circumstances under which each management practice was associated with increased grain yield. Trends in grain yield were sometimes (but not always) related to the variation in lodging between environments and/or seasons.

Application of PGRs induced four main types of response: increased grain yield in fields where lodging was moderate to severe; increased grain yield when lodging was negligible; no grain yield increase when lodging was negligible and rare instances where yield was decreased in response to PGR application. The first two responses (increased grain yield) occurred when sowing soil N levels were greater than 120 kg ha^–1^. The third response (no grain yield effect) was mostly associated with fields where either (i) soil N at sowing was low (i.e. 50–70 kg ha^–1^) and in-crop N application was used to reduce lodging risk, or (ii) experiments were only partially irrigated. The fourth response (negative grain yield response) was rare, occurring in just 2% of comparisons in fully irrigated fields with moderate or high sowing N, and in one of the partially irrigated experiments.

It is important for farmers in the region to understand their management options when growing irrigated wheat on fields with high soil N, such as those of north-eastern Australia where > 200 kg ha^–1^ of N has been frequently observed at sowing (Peake et al., 2014). These soil N levels arise through a combination of residual N from previous crops, and mineralisation of N over the multi-year fallows that can occur due to irregular water supply. Our study found that PGR application had a positive or neutral effect on grain yield in high N environments. The effect was not strictly dependant on the occurrence of lodging, with large yield increases (0.5 t ha^–1^) recorded in response to PGR application in some environments where lodging was negligible. This gives confidence to farmers that PGR application can increase yield and reduce lodging of a wide range of cultivars when soil N at sowing is high. The variable response of cultivars to PGR application was potentially due to variability in the power of statistical analysis between experiments and the difficulty of applying PGRs at a consistent growth stage for each cultivar in each experiment. Precise application of PGRs at the optimum growth stage to individual cultivars may achieve more consistent yield gains than observed herein. In rare instances, a negative yield response was observed in high or moderate N fields. This may have occurred because the increased yield potential associated with PGR use increases weight (and leverage) at the top of the plant (Berry et al., 2004) which can subsequently worsen late season lodging, leading to eventual yield losses. The same mechanism probably explains the increased grainfill lodging that was occasionally observed in association with PGR application.

The increased grain yield we observed in response to PGR application when lodging was negligible has previously been observed in studies using the same PGR-mix at slightly different rates (Matysiak, 2006; Zhang et al., 2017). Similar results have also been observed in studies using chlormequat chloride alone for either winter wheat (Pinthus and Rudich, 1967; Mathews and Caldicott, 1981) or spring wheat (Harris, 1978). However, none of these studies reported both soil mineral N and fertiliser N available to the experimental treatments, and it was not possible to ascertain trends in the literature in relation to impact of N availability on PGR efficacy. Future studies of PGR efficacy should ensure that both soil N at sowing and fertiliser N regime are reported, to allow more detailed assessment of environmental factors influencing yield gains in the presence or absence of lodging.

The mechanism of PGR-associated yield increases in the absence of lodging is also unclear. PGR application to crops during vegetative growth has been reported to reduce height and above-ground biomass for a range of PGR products (Berry et al., 2004). It has been demonstrated that maximum grain yield of winter wheat was obtained by having a moderate canopy density, achieved by reducing N supply (Sylvester-Bradley et al., 1997). In the absence of lodging, PGR application (and the subsequently reduced canopy size) might increase grain yield due to more efficient light interception through the entire canopy (Duncan, 1971; Burgess et al., 2017), or more efficient use of undetermined scarce resources (e.g. micronutrients or water). Further study is necessary to determine the mechanisms causing increased grain yield in response to PGR application, in the absence of lodging.

In the third PGR response category, our results indicated that PGR application rarely increased grain yield in fields where low sowing N (i.e. 50–80 kg soil mineral N ha^–1^) was used in conjunction with in-crop N application to minimise lodging. It is probable that successful implementation of in-crop N application (i.e. canopy management) eliminated the excessive crop canopy size that underpins PGR response. The use of either (but not both) of these practices is therefore recommended for irrigated wheat production on vertosol soils in north-eastern Australia. The fourth PGR response occurred most noticeably when decreased grain yield was observed in a partially irrigated experiment at Brookstead 2013, where water supply was limited, and grain yield was below 6 t ha^–1^. This result contrasted with the results of Barányiová and Klem (2016) who found that chlormequat chloride or trinexapac-ethyl used individually could increase grain yield of winter wheat under water deficit. Their findings are potentially related to the results of De et al. (1982) who found that application of chlormequat chloride could lead to an increase in root:shoot ratio, potentially decreasing the effect of water stress through reduced above ground biomass and a larger root system. However, Green (1986) presented evidence showing both positive and negative yield responses to PGR application in water deficit scenarios. In our partially irrigated experiment, the reduced grain yield associated with PGRs may have been caused by application at a sub-optimal growth stage or an unknown negative interaction between PGR application and the environmental conditions experienced at this particular site.

In-crop N application is commonly used to reduce lodging risk and increase grain yield of high yielding production fields for both spring and winter wheat (Mulder, 1954; Kheiralla et al., 1993; Crook and Ennos, 1995; Berry et al., 2000; Islam et al., 2002; Tripathi et al., 2003; Ercoli et al., 2013; Pampana et al., 2013; Peake et al., 2016). However, the results of the current study demonstrated that G × E × M interaction exists, as the grain yield response was variable between cultivars and location. Cultivars such as Suntop and Cobra had consistently positive grain yield responses to in-crop N application, while Mitch, Lancer, Trojan and Wallup displayed neutral or negative grain yield responses. Additionally, the cultivar Kennedy exhibited increased grain yield and reduced lodging in response to in-crop N application in a warm sub-tropical environment when used without PGRs, but had decreased grain yield in comparison to sowing N application at the same location when PGRs were also used. This result agreed with those above that showed little benefit of PGR application when used in conjunction with in-crop N application on a low N soil.

Interestingly, the four cultivars rated as resistant (Cobra) or moderately resistant to lodging (Suntop, Mitch and Wallup; Peake et al., 2017) were the cultivars that showed the greatest and most consistent grain yield responses to in-crop N application (or in the case of Mitch, a neutral response). Alternatively, two cultivars with greater lodging susceptibility (Trojan and Lancer) displayed decreased grain yield in response to in-crop N application at Spring Ridge 2015 and 2016 where there was negligible lodging.

While in-crop N application has been shown to decrease lodging of susceptible cultivars in severe lodging seasons (Peake et al., 2014), the small lodging reductions achieved by in-crop N application herein may not have been responsible for the grain yield increase in the lodging resistant cultivars. This is particularly evident given that Cobra had increased grain yield in response to in-crop N application, that was not associated with a significant reduction in lodging. It is possible that lodging resistant genotypes possess a canopy structure (e.g. reduced leaf:stem ratio, or a smaller angle between leaf and stem) that interacts with improved late-season N availability to increase grain yield. In-crop N application has previously been advocated in sub-tropical Australia to increase grain yield of irrigated wheat through reduced lodging risk. However, the practice may now be more important for its role in a G × M combination (i.e. in-crop N + lodging resistant cultivars) that increases grain yield of lodging resistant cultivars through improved N availability during the critical period for yield formation. This possibility was also evident in the seminal studies of canopy management in winter wheat production (Sylvester-Bradley et al., 1997, 2000).

It is noteworthy that the in-crop N regimes used across environments and seasons were not identical because soil N at sowing varied noticeably between site/year combinations. Some fields had more N available at sowing than is recommended for the region (50–70 kg N/ha of mineral N + sowing fertiliser N; Peake et al., 2014) which reduces the effectiveness of the in-crop N strategy (Peake et al., 2016). However, it is possible that alternative in-crop N strategies exist which may work effectively for the long-duration cultivars or high levels of sowing N. It is therefore recommended that future studies into optimum N regime should be conducted after first using cover crops to reduce soil N, so that uniform levels of soil N can be achieved across locations. Nevertheless, the results demonstrated the importance of assessing G × E × M interaction though multi-environment testing of multiple genotypes. Optimum N application strategy varies with cultivar and environment, and further research is necessary to determine the optimum N application strategy for new cultivar releases at localities relevant to irrigated wheat production in sub-tropical Australia.

The study also demonstrated the importance of row spacing in managing the balance between lodging risk and increasing yield potential. The complex G × E × M interactions showed that while narrow row spacings were most likely to increase grain yield, some cultivars and management practices could require alternative row spacings to optimize grain yield. When lodging was negligible at a more temperate environment (Spring Ridge), large yield increases were generally achieved by using the narrowest row spacing for all cultivars. This trend was particularly evident for the highest yielding agronomic treatment combination (i.e. PGR application in conjunction with sowing N application). However, when lodging was severe at the same location in 2014, grain yield response across row spacing varied significantly with cultivar and PGR treatment. Application of PGRs in conjunction with the narrowest row spacing was the highest yielding agronomic treatment for most cultivars in this environment, with the exception of the cultivar Merinda which consistently displayed the highest grain yield on the intermediate (28 cm) row spacing. Ongoing testing on newly released cultivars is necessary to determine whether narrow rows in conjunction with PGR application would achieve maximum grain yield, under similarly severe lodging pressure.

In contrast, narrow row spacing did not lead to an increase in grain yield at the subtropical environment, where only short duration cultivars were tested. Our results therefore contrast with the results of Hussain et al. (2013) from a similar latitude (Multan, Pakistan) who found that short statured, low-tillering cultivars had the highest grain yield under irrigation at 10 cm row spacing compared to 20 or 30 cm. Nevertheless, our results are similar to those of Fischer et al. (2005, 2019) also at a similar latitude in the Yaqui Valley (Mexico), who found that cultivars released after the late 1980s could compensate almost completely for a 44 cm gap between outside rows of adjacent raised beds. Our results showed that in the absence of lodging, narrower rows were better suited to take advantage of the greater yield potential and longer growing season available at a more temperate environment, but row spacing did not have an effect on grain yield in a lower yielding, subtropical environment.

The interaction of sowing time and cultivar duration is the subject of considerable research in Australian winter cereal production systems. This is due to the rapid change between seasons that is bordered by the occurrence of damaging frosts just prior to anthesis, and heat stress during grainfilling (Flohr et al., 2017). Studies in both rainfed and irrigated environments have demonstrated that long duration cultivars showed increased grain yield compared to short duration cultivars, when sown at their respective optimum dates to ensure they both reached anthesis during the same optimum flowering window (Coventry et al., 1993; Moore, 2009; Hunt et al., 2015; Flohr et al., 2018; Peake et al., 2018; Hunt et al., 2019). In a study conducted at many of the same irrigated environments used herein, Peake et al. (2018) showed that the yield advantage of long duration cultivars (sown early) was 0.7 t ha^–1^ on average across environments, and up to 1.5 t ha^–1^ in environments that experienced greater levels of water stress. Water stress occurs frequently on commercial irrigated farms where poorly designed infrastructure, labour shortages or mechanical failure can all limit water supply to the crop. Long duration cultivars (sown early) likely developed deeper root systems (Barraclough and Leigh, 1984; Incerti and O’Leary, 1990; Thorup-Kristensen et al., 2009) that allowed them to better withstand the intermittent water stress experienced at some environments. However, Peake et al. (2018) showed that the yield advantage associated with early sowing was smaller or even absent in environments where lodging was more severe. The potential increase in grain yield by sowing early in irrigated production fields must therefore be weighed with the potential for increased lodging. Later sowing has generally been promoted as an effective control method to reduce lodging risk in both winter and spring-wheat production systems (Hanley et al., 1961; Stapper and Fischer, 1990; Spink et al., 2000).

Our results showed that when comparing the same cultivars across two sowing dates, early sowing often (but not always) increased grain yield. The significant cultivar × sowing date × environment interaction for grain yield was partially attributed to differences in cultivar lodging susceptibility. At two of the heavily lodged environments (Narrabri and Spring Ridge 2014), the lodging resistant cultivars Suntop and Cobra showed little difference in grain yield between sowing dates, but the lodging susceptible cultivars (Bellaroi, Caparoi, Kennedy and Trojan) all had significantly decreased grain yield associated with early sowing in at least one of these environments. In contrast, all cultivars had greater yield associated with early sowing at the two low lodging environments (Narrabri and Spring Ridge in 2015), where yield response was more dependent on meteorological conditions during flowering and grainfilling.

Interestingly, later sowing was associated with increased lodging at two locations: at Emerald 2014, where early sowing was associated with a smaller yield advantage than that observed at Narrabri and Spring Ridge 2015; and at Spring Ridge 2014 where most cultivars had lower yield when sown on the early sowing date. Lodging was worse for these late sowing dates because the crops were at an earlier (more lodging susceptible) growth stage than those sown on the early sowing date on the day that thunderstorms occurred. This contrasts with results from the United Kingdom where later sowing almost always reduces lodging risk (Berry et al., 2004). However, the summary of Pinthus (1973) compiled from a range of locations showed that late sowing and early sowing could both reduce lodging depending on environment and germplasm. Rather than recommend one practice or the other, they recommended that ‘adopting a suitable sowing date may contribute to the prevention of lodging’. The results from our study are significant to farmers and researchers in sub-tropical Australia, who should be aware that late sowing may not reduce lodging risk due to the increased frequency of thunderstorms during grainfilling.

It is important to understand that lodging susceptible cultivars are often preferred by farmers due to improved quality traits and/or disease resistance, or sometimes because seed availability is greater. Additionally, management techniques such as in-crop N application, narrow row spacings or early sowing are sometimes unavailable to farmers due to equipment limitations, or environmental influences (e.g. rainfall) that prevent operations from occurring at the optimal time. Ongoing research is necessary to ensure that new cultivar releases are assessed for their lodging susceptibility in combination with the range of agronomic management options available to farmers. Such knowledge will help maximise farm profitability in the context of the G × E × M interaction that exists for grain yield and lodging in high-yielding, spring-wheat production systems.

## Conclusion

The results of our study indicated the existence of significant interaction between cultivar, environment and agronomic practice (G × E × M) for grain yield and lodging in irrigated spring wheat, although some practices were broadly applicable across a range of cultivars. The application of PGRs and the use of narrow row spacing, early sowing and in-crop N application were relatively consistent in improving grain yield when used in optimum combination with other management techniques and/or lodging resistant cultivars. However, grain yield increases were less consistent and decreased grain yield was sometimes observed when in-crop N application was used in conjunction with certain long duration cultivars, or when sowing date (either early or late) increased lodging severity in susceptible cultivars. The optimum agronomic practice for farmers in the region must vary depending on the cultivar they choose to grow, a choice that varies for reasons of local adaptation (e.g. disease pressure), target grain quality specifications and seed availability. Ongoing study of the interaction of future cultivars with the range of management practices available to farmers is therefore imperative to ensure that farmers possess the tools and tactical management options necessary to maximise profitability in variable climates such as those of sub-tropical Australia.

## Data Availability Statement

The datasets generated for this study are available on request to the corresponding author.

## Author Contributions

AP conceived, designed and implemented experiments, interpreted data and prepared the manuscript. KB designed and conducted the statistical analyses and assisted with manuscript preparation. RF assisted with experimental design, data interpretation and manuscript preparation. BD assisted with data analysis and interpretation and manuscript preparation. MG and NP assisted with experimental design and conduct and data interpretation. MM assisted with design and conduct of the statistical analyses.

## Conflict of Interest

The authors declare that the research was conducted in the absence of any commercial or financial relationships that could be construed as a potential conflict of interest.
